# Rectal Perforation Secondary to a Self-Administered Water-Hose Enema: A Case Report and Literature Review

**DOI:** 10.7759/cureus.42244

**Published:** 2023-07-21

**Authors:** Hussain A Al Jabran, Hameed Aljawad, Mohomad Chour

**Affiliations:** 1 Surgery, Almoosa Specialist Hospital, Al Ahsa, SAU; 2 Pathology, Almoosa Specialist Hospital, Al Ahsa, SAU

**Keywords:** acute abdomen, colorectal diseases, functional constipation, enema treatment, rectal perforation

## Abstract

Chronic functional constipation is a common condition that can have a significant impact on a patient’s quality of life and healthcare costs. Hydrostatic enemas are a commonly observed practice among patients with chronic constipation. Rectal perforation is a rare yet serious complication that can be fatal if not diagnosed and treated promptly. Here, we present the case of an elderly lady with Parkinson’s disease who presented with upper rectal perforation after using a hydrostatic enema and was treated with Hartmann’s procedure. This case highlights the importance of having a low threshold for suspecting and diagnosing colorectal perforation in patients presenting with abdominal pain after receiving a hydrostatic enema.

## Introduction

Constipation is a common complaint in older adults and can have a significant impact on a patient’s quality of life, healthcare costs and services, hospital admissions, and surgical procedures. In a study from Saudi Arabia, 25% of respondents to a questionnaire reported having constipation. Constipation was defined as having fewer than three bowel movements per week, along with one or more of the following symptoms: pain during evacuation, hard or lumpy stools, or feeling of incomplete evacuation [1].

Constipation has also been associated with lower quality of life. Several studies have reported that people with constipation have lower scores on measures of physical and mental health, as well as lower levels of satisfaction with their lives [2], making it a medical concern that requires a proper approach and management. One of the most common causes of constipation in older adults is autonomic dysfunction, which is a common symptom of Parkinson’s disease (PD) [3]. Although polyethylene glycol is an effective treatment for constipation in PD patients compared to placebo, many patients continue to experience constipation despite treatment [3].

We have observed that many patients report using self-administered retrograde water-hose enemas at home to relieve their constipation. This practice can lead to colonic perforation, which is a serious condition that can be fatal [4]. Here, we present the case of an elderly patient who developed acute abdominal pain and was found to have an upper rectal perforation a few hours after using a retrograde water-hose enema at home. This case highlights the need for further efforts to evaluate and address this common practice.

## Case presentation

**Figure 1:**
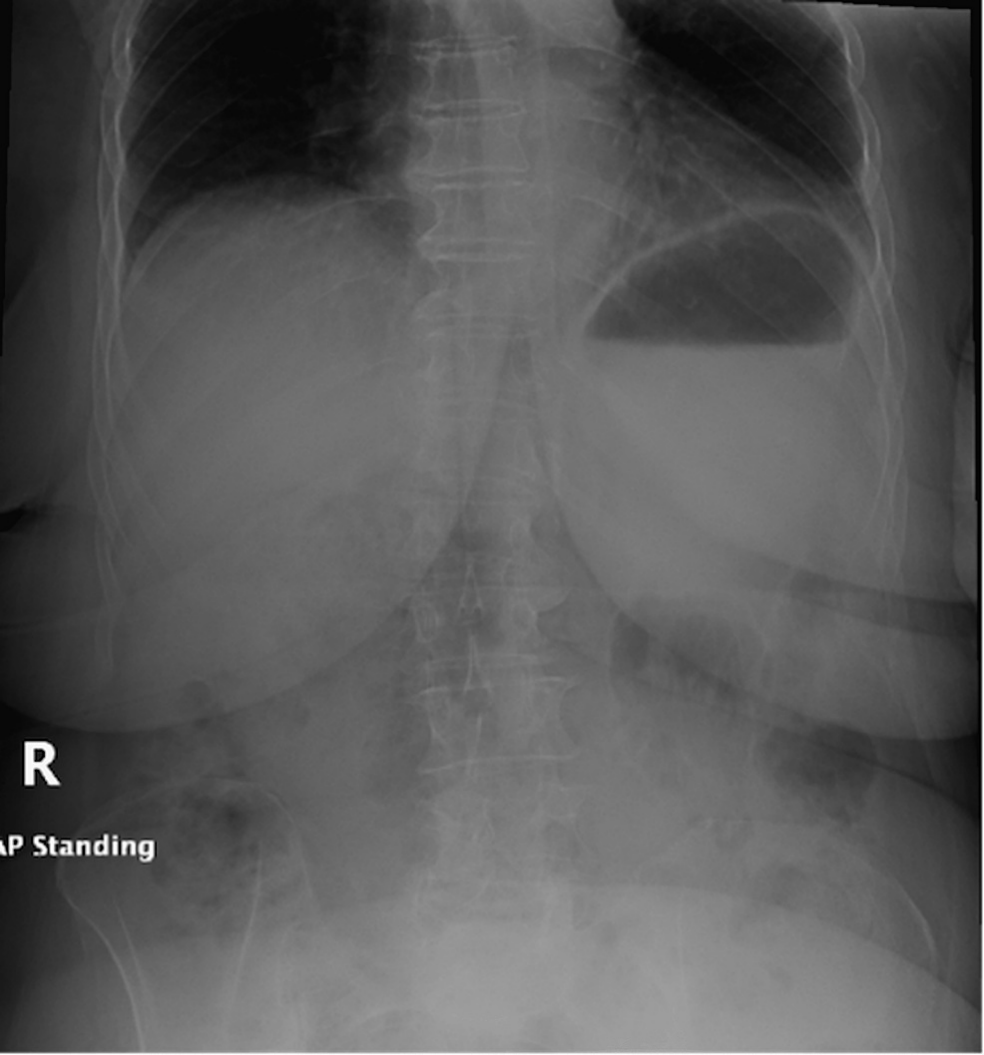
Erect abdomen X-ray showing no air under the diaphragm.

**Figure 2:**
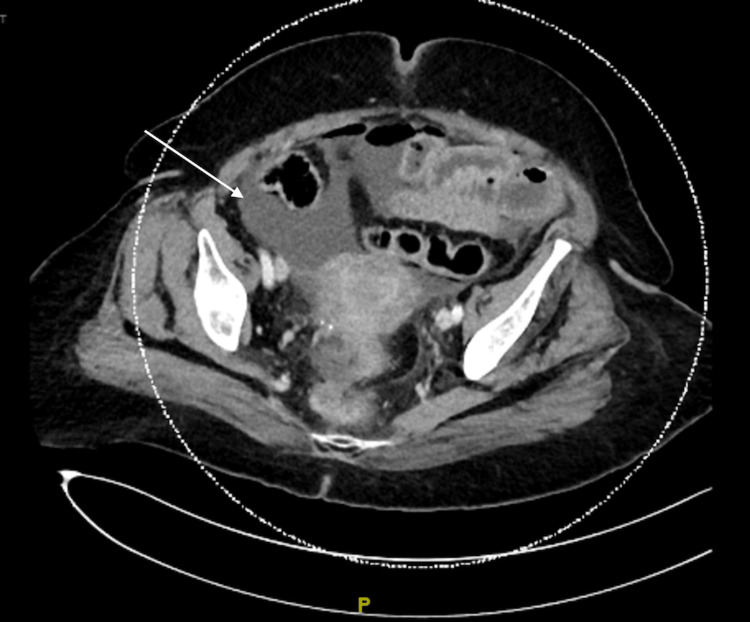
Abdominopelvic CT scan showing moderate peritoneal fluid (white arrow).

**Figure 3:**
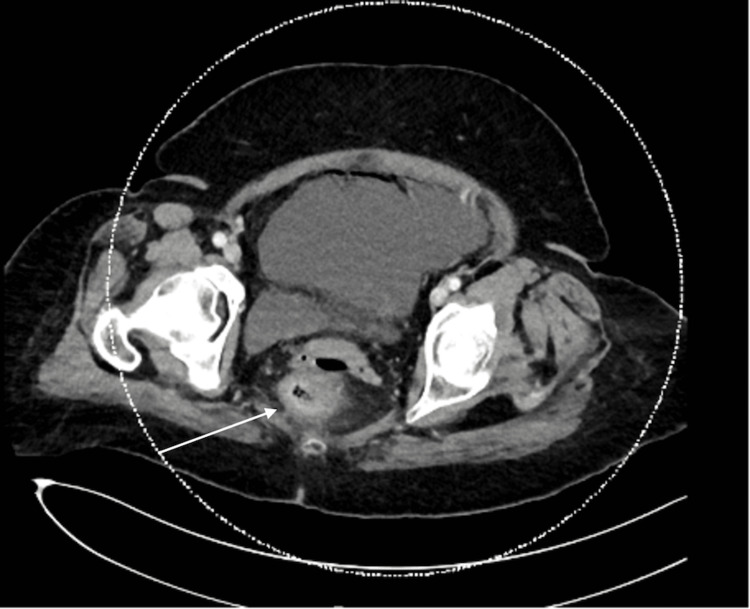
Abdominopelvic CT scan showing diffuse rectal mural enhancement (white arrow) and thickening with mesorectal smudging.

A 77-year-old female with PD on levodopa and amantadine with chronic constipation presented to the emergency department with abdominal pain and distention. She had been experiencing abdominal pain and constipation for one week and had tried a self-administered water-hose enema on the day of the presentation. However, this only made her pain worse, and she developed nausea. On presentation, the patient was afebrile, and her vital signs were within normal limits. Her abdomen was distended and tender to palpation. There was no rebound tenderness or guarding, hepatosplenomegaly, or lymphadenopathy. Laboratory studies showed a white blood cell count of 7,800/μL (normal range = 4,000-11,000/μL). Her electrolytes and liver function tests were normal. On imaging studies, the erect abdomen X-ray was unremarkable (Figure 1). A CT scan of the abdomen and pelvis with contrast showed multiple colonic diverticulosis, diffuse rectal mural enhancement and thickening with mesorectal smudging, scattered intraperitoneal air loculi, and mild-to-moderate free peritoneal fluid (Figures 2, 3).

After the CT scan, the patient’s condition worsened and she started to develop abdominal tenderness and rigidity. She was taken to the operating room for an emergency exploratory laparotomy. The abdomen was full of stool and free fluid. The bowel was examined and an upper rectal perforation extending to the rectosigmoid area was noted. The surgical team performed a Hartmann’s procedure with proximal end colostomy and rectal stump left in the pelvis. The patient was admitted to the intensive care unit for further care and management. She was discharged from the hospital in good condition on postoperative day 15.

**Figure 4:**
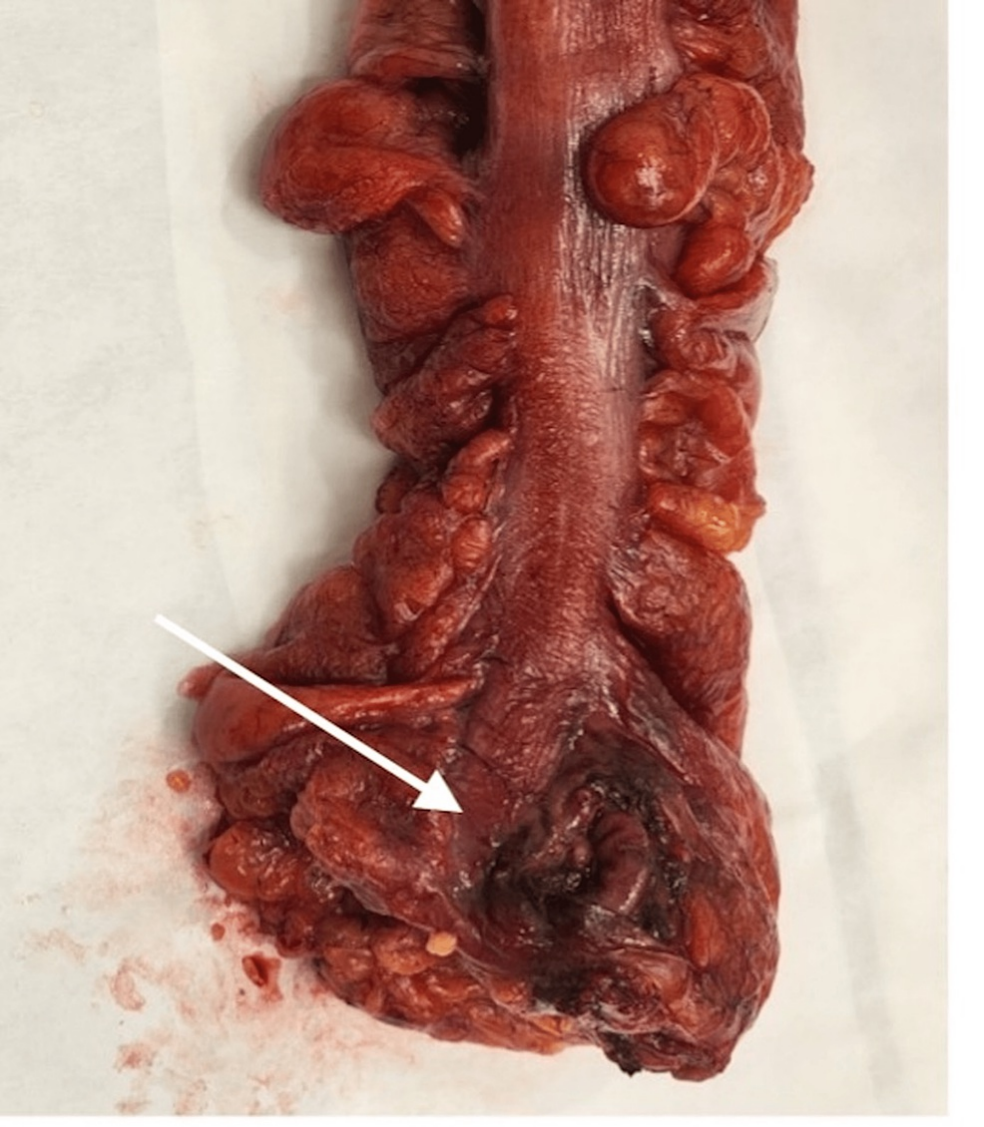
Gross image of the resected specimen with perforation at the upper rectum (white arrow).

**Figure 5:**
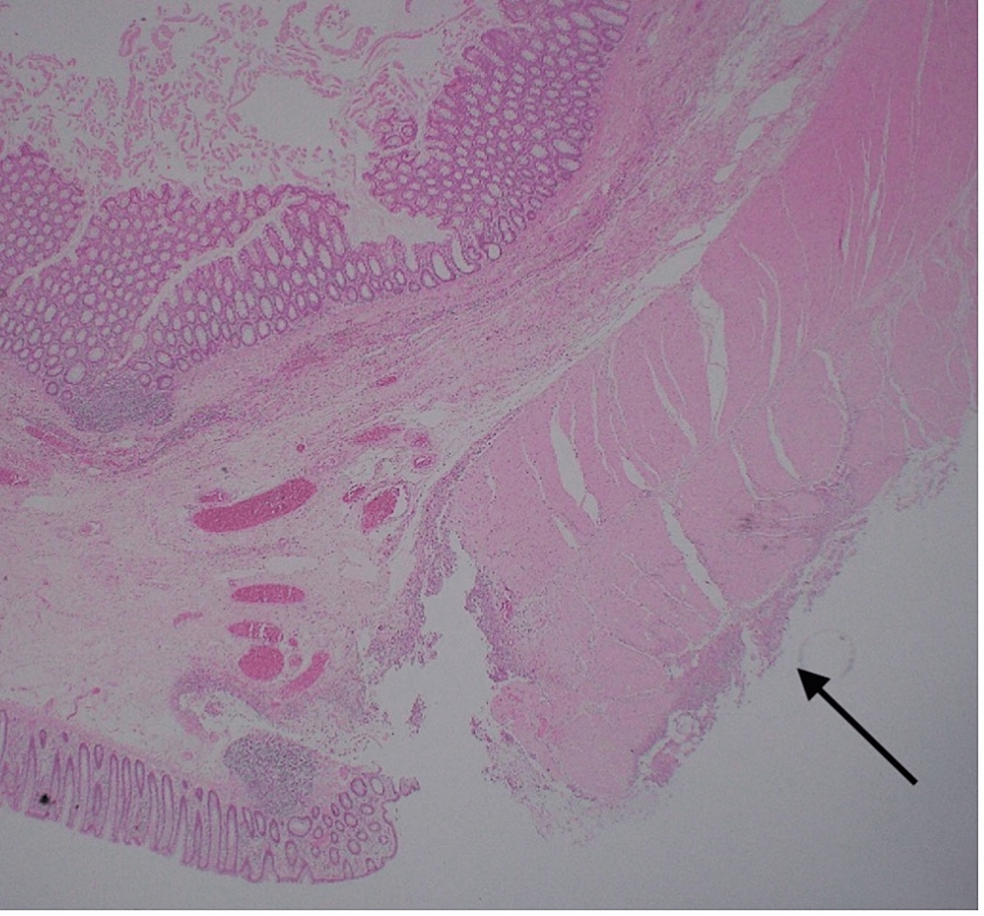
Low-power view of hematoxylin and eosin stain (×2). A representative section of the resected large bowel at the area near the perforation demonstrating benign mucosa and the presence of acute serositis (black arrow).

**Figure 6:**
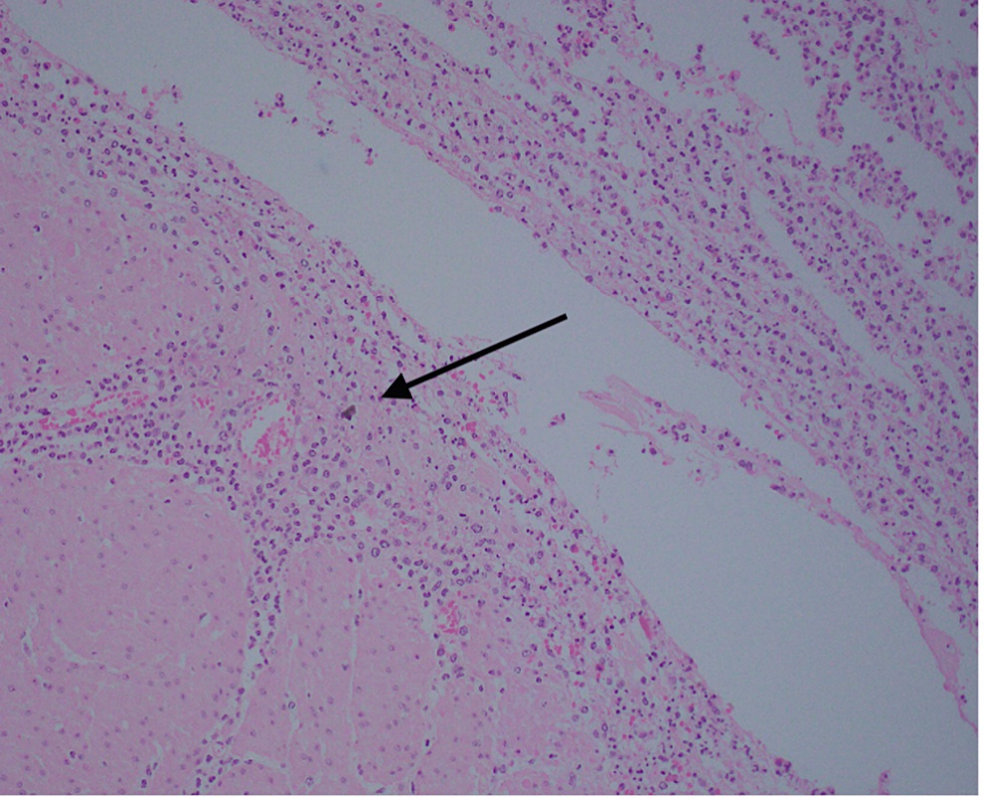
Medium-power view of hematoxylin and eosin stain (×20). The presence of fragments of fecal material (black arrow) on the serosal aspect associated with sheets of neutrophils denoting perforation-associated acute serositis.

The final histopathology showed a segment of the rectosigmoid colon measuring 14.5 cm in length and 6 cm in diameter. A perforation site was grossly identified measuring 1.5 × 1 cm (Figure 4), located 1 cm from the closest peripheral margin. The bowel was opened to reveal unremarkable mucosa except at the perforation site which showed erythema and hemorrhage. No diverticular disease could be identified, and no mucosal tumors were seen. Histological sections revealed benign non-dysplastic colonic mucosa. There were sheets of neutrophils present at the serosal side associated with fragments of fecal debris and vegetable material denoting perforation-related acute serositis (Figures 5, 6).

The patient was followed up in the outpatient department for several months after discharge and was found to be in good health with no stoma-related complications.

## Discussion

Chronic functional constipation is a common problem affecting around 15-17% of adults. It results in 2.5 million physician visits in the United States each year [5], and the annual direct medical costs associated with constipation were estimated to exceed $230 million [6]. The high prevalence, cost, and negative impact on the quality of life and health make constipation a major public health issue [7].

Over 50% of PD patients experience decreased stool frequency (fewer than three bowel movements a week) and 57-67% experience difficult defecation. These rates are significantly higher than those in the general population [8-10]. The mechanism underlying constipation in PD is multifactorial and may include slow colonic transit and anorectal dysfunction [7,11]. Anorectal dysfunction is a condition that affects the muscles and nerves involved in defecation and can cause difficulty in passing stool, straining, and a feeling of incomplete evacuation. Constipation due to slow colonic transit is not related to age, physical activity, or treatment in PD patients [12]. Constipation can have a significant impact on a patient’s quality of life. In some cases, it can lead to serious complications, such as megacolon [13,14], intestinal pseudo-obstruction, volvulus, and bowel perforation [15].

Treatment for constipation in PD patients typically begins with non-pharmacological agents, such as fiber supplements, stool softeners, and probiotic supplements. If these are not effective, pharmacological laxative treatments (bulk, osmotic, and stimulant) may be used. In some cases, newer modalities, such as Lubiprostone (a chloride channel activator), Prucalopride (a 5-HT4 receptor agonist), and Relamorelin (a ghrelin agonist) may also be used [16].

Retrograde rectal enemas are commonly used to treat chronic constipation, especially in the elderly. However, this practice can sometimes lead to rare but serious complications such as bowel perforation. A systemic review and meta-analysis found that most patients who developed rectal perforation after rectal enemas had received the enema from a nurse, followed by self-administrated enemas. Phosphate enemas were the most commonly used type [17].

Recently, there has been an increase in the number of patients who report using self-administered retrograde water-hose enemas (also known as hydrostatic enemas) at home to relieve constipation. Several reports have shown that rectal or colonic perforation is sometimes a possible outcome of this common practice [4]. The clinical presentation of these patients can vary depending on the site of perforation, either colonic or rectal. Rectal perforation is the most common, followed by rectosigmoid and sigmoid colon perforation [17]. The rectum is located in the pelvic cavity, but its anatomical location next to the anorectal complex makes it more susceptible to perforation. The most common symptom of rectal perforation is abdominal pain. Other symptoms may include fever, hematochezia, and back pain [17]. A careful history and physical examination are important for diagnosing rectal perforation, especially in patients who report sudden abdominal pain after using an enema [4].

In this case, the surgery team ordered a CT scan but it failed to demonstrate a clear radiological picture of perforation. Subsequently, they explored the patient’s abdomen based on her history of using a water-hose enema at home and her abdominal findings during the physical examination. It is crucial to maintain a high index of suspicion for this rare but serious complication, as delays in diagnosis are associated with worse outcomes and higher morbidity and mortality [18].

CT scan is a valuable diagnostic tool for confirming the diagnosis of rectal perforation and assessing other causes of acute abdomen. Pneumoperitoneum is usually seen with the perforation of the colon or intraperitoneal part of the rectum, but it is reported less with perforation of the extraperitoneal rectum. In our patient, the perforation was in the intraperitoneal part of the rectum, and a massive pneumoperitoneum is an expected finding in the CT scan. In patients with perforation of the extraperitoneal rectum, the infection may spread more slowly from the perirectal fat to the retroperitoneal space, and a CT scan may show non-specific findings such as mural thickening and mesorectal smudging [17].

The conflict in our case is represented by the discordance between CT radiological findings, on one side, and intraoperative findings and histopathology reports, on the other. As mentioned previously, the perforation was intraperitoneal in origin at the upper rectum which was confirmed by intraoperative findings and the final histopathology report. A massive pneumoperitoneum is an expected finding in the CT scan with such a large intraperitoneal perforation (about 1.5 × 1 cm), but it was not seen in the patient’s CT scan, with the findings being inconclusive in the CT report for clear intraperitoneal perforation. Other CT findings of rectal mural enhancement and thickening can be seen with other pathological processes of the rectum or can represent extraperitoneal perforation of the rectum; however, this was not the scenario in our case. Although a CT scan is a very sensitive tool to evaluate the abdomen and diagnose these cases, sometimes it can be misleading. Physicians must always rely on patient history and physical examination and have a low threshold for diagnosing these cases.

The choice of surgical intervention for patients with acute colorectal perforation depends on several factors [19], including (1) the site of perforation and the clinical stability of the patient. Perforations of the intraperitoneal rectum and colon require different surgical approaches than those affecting the extraperitoneal rectum. (2) The nutritional status. Patients who are malnourished are more susceptible to complications. (3) Medical history. Patients with chronic conditions such as diabetes or heart disease may be at an increased risk for complications after surgery. (4) Chronic medications. Patients taking steroids or other immunosuppressants may be at an increased risk for infection after surgery, or even worse complications such as poor wound healing and anastomotic leak.

The main goals of surgical intervention include (1) cleaning the abdomen (abdominal washout). This is especially important in cases of fecal peritonitis with gross stool contamination. (2) Removing the perforated area of the bowel. This may be done with a primary anastomosis, or with a fecal diversion. (3) Draining the abdomen to prevent further collection and abscess formation.

The specific type of intervention that is used depends on individual patient circumstances and the judgment of the surgeon [19]. Hartmann’s procedure, which involves the resection of the perforated segment of the colon with a proximal end colostomy, has been reported to be the most common procedure used to manage these patients. Other reported treatments include a diverting proximal loop colostomy and sigmoid colon exteriorization [17].

In our case, the surgical team decided to perform Hartmann’s procedure. A primary anastomosis was not attempted due to concerns about the patient’s clinical instability during surgery. It is important to submit the specimen to the histopathology department for further analysis. The histopathology findings can confirm whether or not there is any neoplastic process.

There is a need to raise awareness about the risks of water-hose enemas among the public. Many patients are not aware of the dangers of this practice and may believe that it is a safe and effective way to relieve constipation. It is important to educate people about the risks of water-hose enemas and to encourage them to seek medical treatment for constipation from a qualified healthcare professional.

## Conclusions

Hydrostatic enema is a common practice for patients with chronic functional constipation. A careful history and clinical examination, assisted by imaging studies such as a CT scan with contrast, can help in the early diagnosis of colorectal perforations and improve outcomes. Clinicians should always be on the lookout for this complication, especially in patients who report sudden severe abdominal pain after using a rectal enema. In addition, patients should be educated about the risks of hydrostatic enema.
